# Gemcitabine and doxorubicin in immunostimulatory monophosphoryl lipid A liposomes for treating breast cancer

**DOI:** 10.1002/btm2.10188

**Published:** 2020-09-16

**Authors:** Debra Wu, Zongmin Zhao, Jayoung Kim, Amaya Razmi, Lily Li‐Wen Wang, Neha Kapate, Yongsheng Gao, Kevin Peng, Anvay Ukidve, Samir Mitragotri

**Affiliations:** ^1^ John A. Paulson School of Engineering and Applied Sciences Harvard University Cambridge Massachusetts USA; ^2^ Wyss Institute of Biologically Inspired Engineering Harvard University Boston Massachusetts USA; ^3^ Harvard‐MIT Division of Health Sciences and Technology Massachusetts Institute of Technology Cambridge Massachusetts USA

**Keywords:** chemoimmunotherapy, doxorubicin, gemcitabine, liposomes, monophosphoryl lipid A, triple‐negative breast cancer

## Abstract

Cancer therapy is increasingly shifting toward targeting the tumor immune microenvironment and influencing populations of tumor infiltrating lymphocytes. Breast cancer presents a unique challenge as tumors of the triple‐negative breast cancer subtype employ a multitude of immunosilencing mechanisms that promote immune evasion and rapid growth. Treatment of breast cancer with chemotherapeutics has been shown to induce underlying immunostimulatory responses that can be further amplified with the addition of immune‐modulating agents. Here, we investigate the effects of combining doxorubicin (DOX) and gemcitabine (GEM), two commonly used chemotherapeutics, with monophosphoryl lipid A (MPLA), a clinically used TLR4 adjuvant derived from liposaccharides. MPLA was incorporated into the lipid bilayer of liposomes loaded with a 1:1 molar ratio of DOX and GEM to create an intravenously administered treatment. In vivo studies indicated excellent efficacy of both GEM‐DOX liposomes and GEM‐DOX‐MPLA liposomes against 4T1 tumors. In vitro and in vivo results showed increased dendritic cell expression of CD86 in the presence of liposomes containing chemotherapeutics and MPLA. Despite this, a tumor rechallenge study indicated little effect on tumor growth upon rechallenge, indicating the lack of a long‐term immune response. GEM/DOX/MPLA‐L displayed remarkable control of the primary tumor growth and can be further explored for the treatment of triple‐negative breast cancer with other forms of immunotherapy.

## Graphical abstract

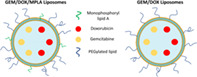

## INTRODUCTION

The engineering of the tumor immune response has rapidly become an integral part of cancer therapies. Treatments such as checkpoint inhibitors have significantly improved patient prognosis in late‐stage non‐small cell lung cancer^1^ and melanoma.^2^ Studies have shown that breast cancer, while traditionally considered immunologically cold,^3^ may also manifest host antitumor immune responses that may be amplified through use of immunotherapy.^4^, ^5^ However, few clinical trials of checkpoint inhibitor monotherapy in the treatment of triple negative breast cancer have demonstrated substantial efficacy.^6^ The mechanisms by which breast cancer cells escape immune recognition are still not fully recognized, but include recruitment of suppressive immune cells such as regulatory T cells and tumor‐associated macrophages, as well as the secretion of immune inhibitory cytokines.^7^ Breast cancer subtypes also express relatively low levels of tumor antigens, which makes recognition difficult for activated cytotoxic T‐cells.^8^

The use of immune adjuvants to boost recognition of otherwise poorly immunogenic antigens can potentially improve the immune microenvironment of breast cancer. Clinically approved immune adjuvants include oil/water emulsions, aluminum salts, and agents that activate innate immunity by binding to “Toll”‐like receptors (TLRs) that recognize pathogen‐associated molecular patterns.^9^ One such adjuvant, monophosphoryl lipid A (MPLA), is a detoxified derivative of lipopolysaccharide (LPS) from *Salmonella minnesota* R595. MPLA was the first TLR adjuvant approved for clinical use and is currently licensed for use in Ceravix (human papilloma virus‐16 and ‐18 vaccine) and Fendrix (Hepatitis B vaccine).^10^ MPLA has also been incorporated in liposomes in the malaria vaccine AS01E (or AS01B) and was shown to induce stronger cytotoxic T cell reactions than formulations that had similar composition but smaller particle size.^11^

Recent work has shown MPLA to be effective in altering the tumor immune environment when used in liposomes containing immune stimulating cytokines.^12^ MPLA may also sensitize breast cancer tumors to doxorubicin (DOX) treatment.^13^ However, the effect of MPLA in combination with different drug pairs has not been extensively explored. The immune effects of chemotherapy have long been disregarded, as drug cocktails were administered to the point of patient myelosuppression.^14^ Also, human‐derived tumor cell lines are typically implanted in immunodeficient mouse models to ensure tumor growth, resulting in the development of most chemotherapy combinations without consideration of immune effects. However, in the past decade focus has shifted to understanding the immune interactions of low‐dose chemotherapy with immunotherapy, and the identification of immunogenic chemotherapy combinations that can enhance immune responses.^15^, ^16^, ^17^, ^18^

We have recently shown very effective tumor control with gemcitabine (GEM) and DOX liposomes in the orthotopic 4T1 murine breast cancer tumor model.^19^ GEM and DOX, both commonly used chemotherapeutics, were co‐loaded into liposomes with lipid content representative of clinically used formulations. DOX has been reported to stimulate immunogenic cell death of tumor cells, prompting immune recognition and activation,^20^ and GEM has been shown to restrict myeloid‐derived suppressor cells while promoting antigen cross‐presentation in dendritic cells.^21^ Treatment with the co‐loaded liposome in the 4T1 murine breast cancer tumor model produced a moderate response in terms of increased M1/M2 macrophage ratio in the tumor immune infiltrate. In this work, we incorporated MPLA into the lipid bilayer of GEM/DOX liposomes and evaluated the benefit of MPLA addition in terms of immune response and overall efficacy. Our results show that GEM/DOX MPLA liposomes induced a strong effect on the growth of primary tumor. MPLA produced short‐term immune activation benefits but did not lead to a long‐term immune response upon tumor rechallenge. However, the short‐term dendritic cell activation, along with the strong effect on the primary tumor, may make GEM/DOX/MPLA liposomes suitable for combination with other forms of immunotherapy to better treat triple‐negative breast cancer.

## MATERIALS AND METHODS

### Liposome fabrication and cell culture materials

1,2‐distearoyl‐sn‐glycero‐3‐phosphocholine (DSPC) and 1,2‐distearoyl‐sn‐glycero‐3‐phosphoethanolamine‐*N*‐[methoxy(polyethylene glycol [PEG])‐2000] (DSPE‐mPEG2000) were purchased from Avanti Polar Lipids (Alabaster, AL). Cholesterol was purchased from Millipore Sigma (Burlington, MA). MPLA from *Salmonella enterica* serotype minnesota Re 595 was purchased from Millipore Sigma. Doxorubicin hydrochloride was purchased from LC labs (Woburn, MA) and gemcitabine hydrochloride was purchased from Oxchem Corporation (Wood Dale, IL).

4T1 murine breast cancer cells (ATCC CRL‐2539) and JAWSII immature murine dendritic cells (ATCC CRL‐11904) were purchased from ATCC (Manassas, VA). 4T1 cells were grown in RPMI‐1640 supplemented with 10% fetal bovine serum (FBS) and 1% penicillin/streptomycin. JAWSII dendritic cells were grown in alpha‐MEM supplemented with 20% FBS, 1% penicillin/streptomycin, 4 mM l‐glutamine, and 5 ng/ml granulocyte‐macrophage colony‐stimulating factor. Cellular inhibition assays used 3‐(4,5‐dimethylthiazol‐2‐yl)‐2,5‐diphenyltetrazolium bromide (MTT) to quantify cell viability. All materials were purchased from ThermoFisher Scientific (Waltham, MA). Cell culture flasks and tissue culture‐treated well plates were purchased from Corning (Corning, NY).

### Tumor model and flow cytometric analysis materials

All animals used were female BALB/c mice (age 50–56 days) purchased from Charles River Laboratories (Wilmington, MA). Heparin‐coated plasma preparation tubes, Gibco™ Type 1 Collagenase, ACK Lysing Buffer, Invitrogen™ UltraComp eBeads™ Compensation Beads, and SYTOX™ Blue Dead Cell Stain were also purchased from ThermoFisher Scientific. DNAse I was purchased from Roche (Indianapolis, IN). Cell staining buffer was purchased from Biolegend (San Diego, CA). Round‐bottom 96 well plates were purchased from Corning. Antibodies (Table S1) were purchased from ThermoFisher Scientific, Abcam (Cambridge, MA), and Biolegend.

### GEM/DOX liposome fabrication

Liposomes (40 μmol, molar ratio 56.4% DSPC, 5.3% DSPE‐mPEG2000, 38.3% cholesterol) were made by the conventional thin‐film hydration method. When making MPLA liposomes, 0.5 mg of MPLA was incorporated as well. The lipids were dissolved in chloroform and added to a dry round‐bottom flask. The lipids were dried under reduced pressure and heating to produce a thin lipid film. The lipids were then resuspended using 75 mg/ml GEM in 1.1 ml of ammonium sulfate buffer (250 mM, pH 5.5) and hydrated at 70°C for 30 min, followed by extrusion through a 50 nm polycarbonate membrane to create liposomes of similar size. Then, a pH gradient was created through the removal of extra‐liposomal ammonium sulfate salts and unencapsulated GEM by PD‐10 size exclusion columns from GE Healthcare (Chicago, IL). The pH gradient served to actively load DOX (20 mg/ml, 50 μl) at 65°C for 30 min. During this step, 100 μl of 95 mg/ml GEM was also added to reduce GEM loss from diffusion. Then, unencapsulated drugs were removed once more by size exclusion chromatography.

### Liposome characterization

Samples were diluted 10‐fold in 9:1 methanol: water with 0.05% trifluoroacetic acid. After a brief sonication, MPLA was detected by reverse phase HPLC. A Zorbax 300Extend C18 3.5 μm column (150 mm × 4.6 mm) purchased from Agilent (Santa Clara, CA) was equilibrated with 0.5 ml/min 40% mobile phase A (1 mM ammonium acetate) and 60% mobile phase B (2‐propanol, LC–MS grade). Ten microliters of sample was injected using this solvent composition. The solvent gradient gradually changed to become 100% mobile phase B at 15 min. It was then changed back to 60% mobile phase B and 40% mobile phase A at 20 min and was maintained until the end of the run at 25 min. MPLA eluted at approximately 16 min and was detected by UV absorption at 240 nm.

Liposomal size and zeta potential were measured by dynamic light scattering using a Malvern Zetasizer. Size was obtained from the number distribution. In order to detect drug content, samples were diluted 10‐fold in 1:1 methanol: acetonitrile with 0.05% trifluoroacetic acid (*n* = 3). Samples were then sonicated in a water bath for 30 min and centrifuged for 5 min. Sample supernatant was then analyzed for drug concentration by reverse phase HPLC. The Zorbax column used previously in the detection of MPLA was equilibrated with 0.5 ml/min 99% mobile phase A (water with 0.1% trifluoroacetic acid) and 1% mobile phase B (acetonitrile with 0.1% trifluoroacetic acid). Sample (10 μl) was injected at this composition. After injection, the gradient changed to 60% mobile phase B at 10 min. The solvent composition reverted to 1% mobile phase B at 15 min and was maintained until the end of the run at 20 min.

Liposomal release was measured using Amicon Ultra mini dialysis filters purchased from Millipore Sigma. A 10‐fold dilution (100 μl) of the liposomes was placed into the mini dialysis filter (*n* = 5), which was installed over a reservoir of PBS. Samples were kept under constant shaking at 37°C. At each timepoint, the PBS reservoirs were replaced to maintain sink conditions. Released drug was quantified using the drug detection HPLC method described previously.

### In vitro cellular assays

Cells for antibody staining and flow cytometry studies were plated in 6‐well plates in 3 ml of media and allowed to adhere overnight. In single‐cell experiments, 9 × 10^5^ of either JAWSII dendritic cells or 4T1 murine breast cancer cells were plated in 6‐well plates. In co‐culture experiments, 9 × 10^5^ cells consisting of a 1:1 ratio of JAWSII dendritic cells and 4T1 murine breast cancer cells were plated. Treatment was administered approximately 24 h after plating. Cells were harvested using 0.5 ml of trypsin and resuspended to establish 10^6^ cells in 100 μl of cell staining buffer. Cells were washed once and incubated at room temperature with 1% CD16/32 in 100 μl of cell staining buffer. After another wash, cells were incubated for 30 min on ice with fluorescently labeled antibodies (Table S1) to distinguish tumor antigens or characteristic markers of immune cell subtypes. Antibody‐stained cells were then washed twice before analysis with a BD LSRII flow cytometer.

### Tumor model development and treatment

Tumors were developed by injection of 10^5^ 4T1 cells in PBS above the fourth mammary fat pad in female BALB/c mice. Tumors were monitored every other day through caliper size measurements. When tumors were approximately 50 mm^3^, which occurred approximately 7 days after injection, tumors were treated with two intravenous injections of liposomal formulations occurring 4 days apart. Tumors were harvested for immune profiling 48 h after the last treatment.

Treatment efficacy was evaluated with the same tumor implantation procedure. Liposomal formulations were administered when tumors were ~15 mm^3^. Treatment was administered on day 5, 9, and 16 after tumor inoculation. Tumor volume and mice weight were monitored every other day until the control group tumors reached the endpoint criteria of 1000 mm^3^, at which point the study was terminated and tumors were extracted for mass measurements. Mice body weight loss greater than 15% was also a criterion for euthanasia.

In performing the tumor rechallenge, tumors were established with the same implantation procedure. When tumors were ~15 mm^3^ in size, two injections of liposomal formulations were administered 4 days apart. Tumors were observed for ~20 days, at which point 10^5^ 4T1 cells in PBS were injected in the opposite mammary fat pad. Mice were monitored for tumor growth and weight loss.

### Tumor dissociation and immune profiling

Two days after the second administration of treatment, 4T1 tumors were extracted and weighed. Each tumor was cut into small pieces and enzymatically digested using Collagenase Type I (5 mg/ml) and DNAse I (50 U/ml) in 5 ml of HBSS buffer at 37°C for 60 min. Afterwards, the cells were passed through 70 μm cell strainers with trituration and then centrifuged and resuspended in ACK red cell lysis buffer for 2 min at room temperature. The cells were then resuspended in PBS with 50 U/ml DNAse with volume adjusted to obtain 10^6^ cells/ml. One hundred microliters of the cell suspension for each tumor was pelleted and treated with blocking buffer for 30 min at room temperature in a round‐bottom 96 cell plate. Blocking buffer was made by supplementing cell staining buffer (1× PBS, 3% FBS, 30 μM EDTA) with 1% CD16/32. After washing the cells once with cell staining buffer, the tumors were treated with cell marker staining antibodies (Table S1). Leukocytes were identified by CD45, and cells of the myeloid lineage were identified by CD11b. Macrophages were identified by CD11b^+^F4/80^+^ and further differentiated by CD80 (M1) and CD206 (M2). Dendritic cells were identified by CD11b^+^CD11c^+^. Finally, cells were washed twice more in cell staining buffer and subsequently analyzed by a BD LSRII flow cytometer manufactured by BD (Franklin Lakes, NJ) and all data was analyzed with FCS Express 6 software (De Novo Software, Glendale, CA).

### Statistical analysis

Statistical comparison of groups was done using a one‐way analysis of variance with Tukey's multiple comparison test and Student's t‐test in GraphPad Prism v5. Statistical significance was defined as *p < 0.05, **p < 0.01, ***p < 0.001.

## RESULTS

### Liposome fabrication

Liposomes were fabricated by the conventional thin‐film hydration technique and loaded with an equimolar ratio of GEM and DOX. MPLA was incorporated into the lipid bilayer during creation of the thin lipid film. Liposomes are hereafter referred to by their encapsulated agents, and denoted by ‐L. Drug loading, evaluated by HPLC, showed equimolar loading of GEM and DOX achieved with active loading of DOX and passive loading of GEM. The liposomal size and zeta potentials were very similar to that of standard DOX liposomes, representative of clinically used Doxil®.^22^ Additionally, MPLA was quantified as 88.5 μg/ml in the final liposomal formulation. This resulted in a 17.7% encapsulation efficiency and was due to dilution of the liposomes during drug loading and size‐exclusion separation processes. The encapsulation efficiency of GEM and DOX remained similar to previously reported values.^19^ The size and zeta potential of the formulations remained similar, showing that incorporation of a small amount of MPLA does not significantly change the liposome physical properties (Table 1).

**TABLE 1:** GEM/DOX MPLA characterization

	Molar ratio (GEM:DOX)	MPLA (μg/ml)	Size (nm)	Zeta potential (mV)	PDI
DOX‐L	‐	‐	75.5 ± 2.8	−23.3 ± 1.2	0.05 ± 0.02
GEM/DOX‐L	0.8	‐	72.3 ± 2.3	−25.6 ± 1.5	0.09 ± 0.01
GEM/DOX/MPLA‐L	1.0	88.5	72.0 ± 2.1	−26.3 ± 1.4	0.05 ± 0.02

### In vitro cellular activation

**FIGURE 1:**
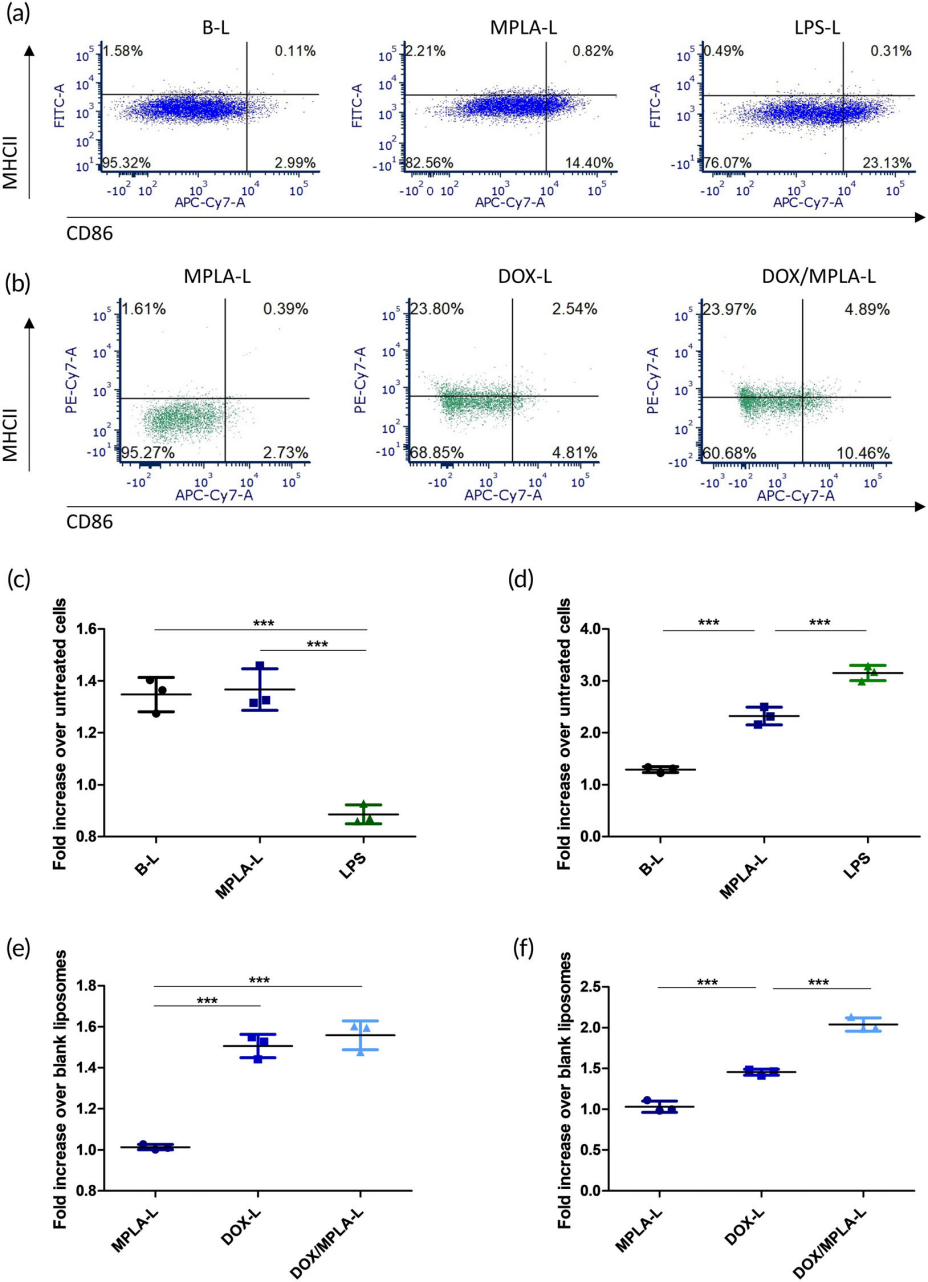
In vitro activation of JAWSII cells alone and in co‐culture with 4T1 cells. Experiments were conducted in triplicate wells, and quantification is displayed as fold increases in mean fluorescence intensity compared to untreated control JAWSII cells (parts (c) and (d)) or equivalent blank liposome treatment in the 4T1 and JAWSII co‐culture (parts (e) and (f)). (a) Representative shift of JAWSII cells treated with blank liposomes (B‐L), MPLA liposomes (MPLA‐L) and LPS. (b) Representative shift of JAWSII cells in co‐culture with 4T1 cells, after treatment with MPLA‐L, DOX‐L, and DOX/MPLA‐L. (c) MHCII expression in JAWSII cells. (d) CD86 expression in JAWSII cells. (e) MHCII expression in 1:1 4T1:JAWSII co‐culture. (f) CD86 expression in 1:1 4T1:JAWSII co‐culture

MPLA has been shown to increase dendritic cell activation.^23^, ^24^ Both blank liposomes and liposomes with ~5 μg/ml MPLA were administered to JAWSII immature murine dendritic cells. 1 μg/ml of liposaccharides (LPS) was used as a positive control for dendritic cell activation. The amount of LPS used was lower than the amount of MPLA because LPS is highly stimulating and a potential cause of decreased cellular viability.^25^ In JAWSII cells, addition of MPLA‐containing liposomes (denoted MPLA‐L) did not cause a significant difference in major histocompatibility complex II (MHCII) expression when compared to treatment with an equivalent volume of blank liposomes (denoted B‐L) (Figure 1a,c). However, there was a significant increase in CD86 expression in groups treated with MPLA‐L compared to blank liposomes (Figure 1a,d), indicating greater dendritic cell activation.

In addition to the immunogenic effects of MPLA, DOX has been shown to increase tumor immunogenic cell death through a variety of mechanisms including the exposure of calreticulin, which stimulates dendritic cell antigen presentation.^20^ A 1.8‐fold increase in calreticulin exposure on 4T1 cells was observed after treatment with 10 μM free DOX compared to untreated controls and increased to approximately threefold upon combination treatment of DOX and liposomes (Figure S1a). There was no significant difference between the free DOX + blank liposomes and free DOX + MPLA‐L, indicating that the inclusion of MPLA does not influence calreticulin exposure. Representative gating for this study is shown in Figure S2.

A co‐culture of both JAWSII cells and 4T1 cells was developed to study dendritic cell activity in the presence of 4T1 cells, which are shown to undergo immunogenic cell death from exposure to DOX.^26^ The 1:1 co‐culture was treated with MPLA‐L, DOX‐L, and DOX/MPLA‐L. As GEM is not reported to stimulate expression of immunogenic cell death markers, GEM‐L and GEM/MPLA‐L were not included in this study.^15^ The co‐culture observed little to no increase in MHCII expression with treatment by MPLA‐L alone, possibly due to immunosuppressive signaling produced by 4T1 cells, such as the production of TGF‐β and IL‐6.^27^ However, DOX/MPLA‐L treatment resulted in a 1.6‐fold increase in MHCII expression (Figure 1b,e) and a twofold increase in CD86 expression (Figure 1b,f). Another co‐stimulatory ligand, CD40, experienced a 2.9‐fold increase in expression when treated with DOX/MPLA‐L (Figure S1b). Representative gating of this experiment is reported in Figure S3.

### In vitro comparison of liposomal toxicity and release profile

**FIGURE 2:**
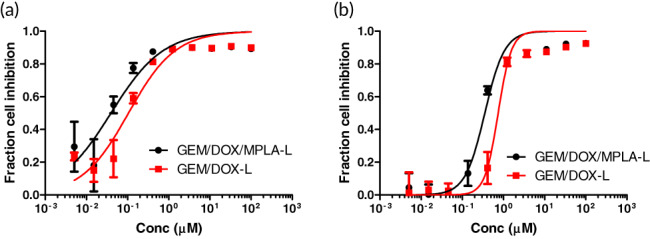
GEM/DOX‐L and GEM/DOX/MPLA‐L in vitro toxicity on 4T1 cells. Both treatments displayed similar dose–response behavior on two different seeding densities. Error bars represent standard deviation with *n* = 6. (a) 500 4T1 cells/well. (b) 5000 4T1 cells/well

GEM/DOX liposomes containing MPLA (GEM/DOX/MPLA‐L) were synthesized and compared to GEM/DOX liposomes without MPLA (GEM/DOX‐L) in terms of in vitro cytotoxicity and release profile. The drug combination was previously shown to possess no synergistic effects using the Combination Index on 4T1 cells.^19^ As MPLA is primarily an immune adjuvant, there was no anticipated effect on 4T1 cells in vitro. Liposomal IC_50_ and hill coefficient derived from the dose–response Hill equation fitted to cellular viability of 4T1 cells plated at 500 cells/well (Figure 2a) and 5000 cells/well (Figure 2b) had no significant differences between the two treatments (Table 2). The IC_50_ of GEM/DOX‐L and GEM/DOX/MPLA‐L increased 6.8‐fold and 8.8‐fold respectively when comparing values from the 500 cell/well and the 5000 cell/well experiments. However, the Hill coefficient of the drug combinations increased to >1 in the 5000 cell/well experiment. The Hill coefficient is an indicator of dose–response curve steepness and can indicate cooperative binding to cell ligands, which may lead to reduction of drug resistance.^28^ This indicates that while there may be a higher drug concentration threshold to surpass in the case of higher tumor burden, the potency of the drug combination is not lost as high Hill coefficient shows effective tumor control once that threshold is met.

**TABLE 2:** Dose–response parameters of GEM/DOX‐L and GEM/DOX/MPLA‐L

500 cells	IC_50_ (μM)	Hill coefficient
GEM/DOX‐L	0.11 ± 0.01	0.77 ± 0.06
GEM/DOX/MPLA‐L	0.04 ± 0.01	0.69 ± 0.07

5000 cells	IC_50_ (μM)	Hill coefficient
GEM/DOX‐L	0.75 ± 0.05	2.70 ± 0.32
GEM/DOX/MPLA‐L	0.35 ± 0.03	1.82 ± 0.26

**FIGURE 3:**
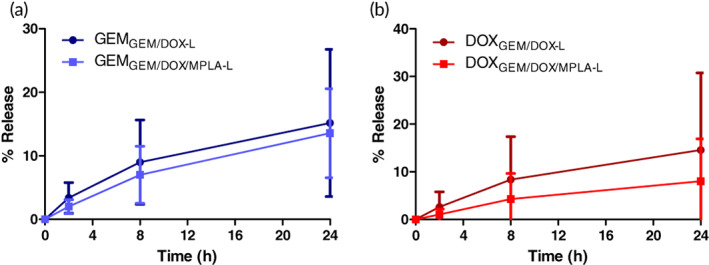
Release profile of liposomal formulations over 24 h at 37°C in PBS. Error bars represent *n* = 5, and statistical significance between groups was measured using Student's *t* test. (a) GEM release. (b) DOX release

Comparable in vitro toxicity of the liposomal formulations is also an indicator of similar release profiles. The release profile of the formulations into PBS was studied for 24 h at 37°C under constant shaking to determine if incorporation of MPLA caused significant deviations in drug release. Comparisons between the release of GEM in both GEM/DOX‐L and GEM/DOX/MPLA‐L showed no significant difference (Figure 3a) and neither did the release of DOX from both formulations (Figure 3b). Furthermore, both formulations showed similar release rates of both encapsulated drugs. GEM/DOX‐L demonstrated stable encapsulation of drugs with ~15% of both drugs released at the end of the 24 hr period (Figure S4a). GEM/DOX/MPLA‐L showed similar stable encapsulation, with 14% of GEM released and 8% of DOX released (Figure S4b). No statistical difference was found between GEM release and DOX release in each formulation. Therefore, MPLA incorporation in the liposomal bilayer did not have a detrimental effect on sustained drug release.

### In vivo efficacy and immune profiling

The liposomal formulations were next evaluated in vivo for immunogenicity and tumor response in the highly aggressive orthotopic 4T1 model. The 4T1 model is also regarded as immunologically cold, making it representative of human breast cancers.^29^ The liposomal formulations were injected twice intravenously at a dosage of 3 mg/kg DOX and 1.55 mg/kg GEM before tumors were extracted 48 h after the final injection. At that dosage, the GEM/DOX/MPLA‐L group delivered a total of 5.7 μg MPLA per injection, which is similar to dosages used in intratumoral injections.^12^, ^30^

**FIGURE 4:**
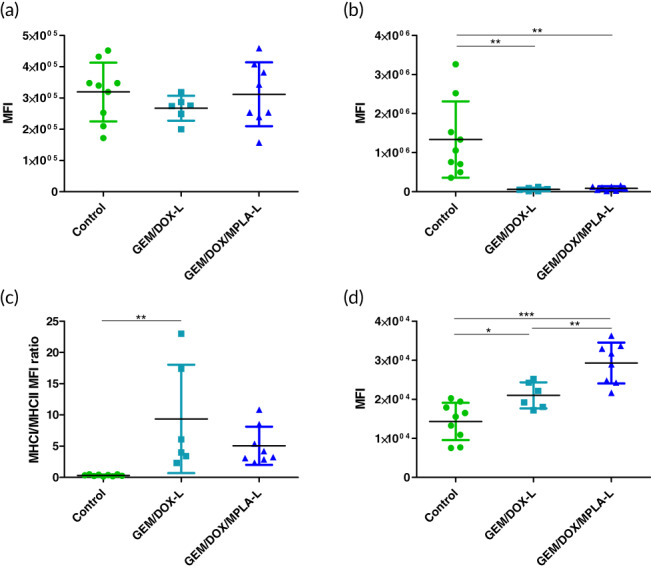
Immune profiling of 4T1 tumors showed increased dendritic cell activation. Expression levels are shown using mean fluorescent intensity. (a) MHC I expression. (b) MHC II expression. (c) MHC I/MHC II ratio. (d) CD86 expression

Dendritic cell activation was studied as the fold change in median fluorescence intensity of each treatment group in comparison to the untreated control group. Expression of major histocompatibility complex I (MHC I) (Figure 4a) and MHC II (Figure 4b) had no significant difference in expression levels between the treatment groups. MHCII expression was significantly lower in the treatment groups compared to the untreated control group. However, the ratio of MHCI to MHCII expression was significantly elevated in GEM/DOX‐L treated mice compared to the control group (Figure 4c). Antigen presentation by MHC class I molecules has proved essential for recognition by T cell receptors on CD8^+^ T cells.^31^ Dendritic cell co‐stimulatory ligand CD86 was significantly upregulated in the GEM/DOX/MPA‐L treatment group (Figure 4d).

**FIGURE 5:**
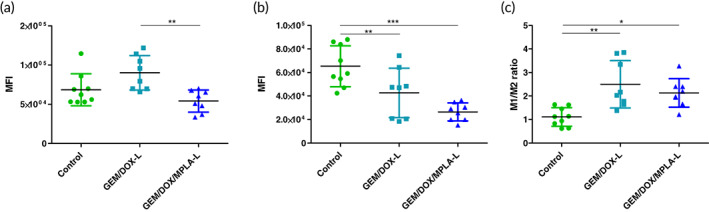
Macrophage population within 4T1 tumors as determined by flow cytometry immune profiling. (a) CD80^+^F4/80^+^ M1 macrophages. (b) CD206^+^F4/80^+^ M2 macrophages. (c) M1/M2 ratio

Immune cell populations in the 4T1 tumor environment were quantified by fluorescent antibody staining and analyzed with flow cytometry. The immune effects of the GEM/DOX combination have been shown in previous work to increase macrophage M1/M2 ratio without impacting the adaptive immune response.^19^ Similar results were observed in this study. While GEM/DOX‐L exhibited increased amounts of CD80^+^F4/80^+^ M1 macrophages (Figure 5a) and both treatment groups exhibited decreased CD206^+^F4/80^+^ M2 macrophages (Figure 5b), there was ultimately no significant difference between M1/M2 ratio between treatment groups, although both were significantly higher than the control group (Figure 5c). Negligible differences in CD11c^+^CD11b^+^ dendritic cells and Ly6G^+^CD11b^+^ myeloid‐derived suppressor cells were found between the GEM/DOX‐L and GEM/DOX/MPLA‐L treatment groups (Figure S5). Representative gating of in vivo dendritic cells and macrophages are given in Figure S6, and the cell populations of dendritic cells and macrophages given as a percentage of total cells can be found in Figure S7. Chemotherapy‐treated groups had lower populations of immune cells, although there was no significant difference between the macrophage count of the GEM/DOX‐L treated group and the control group. Representative gating of Ly6G^+^CD11b^+^ myeloid‐derived suppressor cells is given in Figure S8.

Also, the mass of GEM/DOX‐L and GEM/DOX/MPLA‐L‐treated tumors was significantly less than that of untreated controls, despite undergoing treatment twice with extraction 48 h after the last dosage (Figure S9).

**FIGURE 6:**
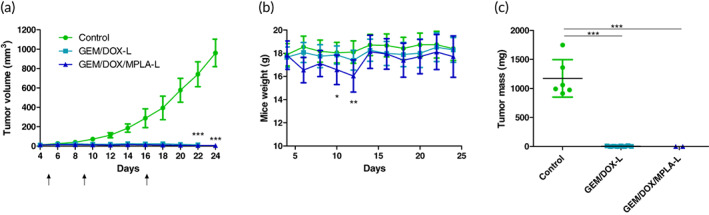
Treatment efficacy of GEM/DOX/MPLA‐L and GEM/DOX‐L in an orthotopic 4T1 tumor model. Three injections of 100 μl of 0.54 mg/ml DOX and 0.28 mg/ml GEM were injected, which translates to 3 mg/kg DOX and 1.55 mg/ml GEM. Mice treated with GEM/DOX/MPLA‐L received 5.7 μg MPLA per injection. (a) Tumor volume measurements, with control (*n* = 8), GEM/DOX‐L (*n* = 8), and GEM/DOX/MPLA‐L (*n* = 5). Difference in *n* arises from fully regressed tumors, which were removed from the tumor volume measurements. Significance is displayed for the final tumor size of both treatment groups in comparison to the control group. (b) Mice weight measurements for GEM/DOX‐L, GEM/DOX/MPLA‐L, and control group. Reported significance is for the GEM/DOX/MPLA‐L group relative to the control group. (c) Tumor mass after extraction on day 27 of study, with control (*n* = 6, due to prior euthanasia of two mice), GEM/DOX‐L (*n* = 8), and GEM/DOX/MPLA‐L (*n* = 2) due to absence of tumors

To measure tumor efficacy, treatment was administered when tumors were approximately ~15 mm^3^ in size. Treatmentcomprised of three injections on days 5, 9, and 16 of GEM/DOX‐L and GEM/DOX/MPLA‐L, both containing 3 mg/kg DOX and 1.55 mg/kg GEM. Mice treated with GEM/DOX/MPLA‐L received 5.7 μg MPLA per injection. Tumors were then monitored until the control tumors reached approximately 1000 mm^3^. The liposomal formulations demonstrated extremely efficient tumor control (Figure 6a). The 4T1 tumor model is known for aggressive growth and lung metastasis. However, both the GEM/DOX‐L and GEM/DOX/MPLA‐L formulations managed to limit tumor growth to under 25 mm^3^. Also, the given dosage of DOX and GEM in co‐loaded liposomes was reduced compared to the doses of either drug alone reported in the preclinical literature,^32^, ^33^ and the dosing schedule allowed for relative stability in mice weight. However, on day 12, mice treated with GEM/DOX/MPLA‐L demonstrated significantly more weight loss (*p < 0.05) than those treated with the purely chemotherapeutic formulation, which were not significantly different in weight from the control group (Figure 6b). One of the mice treated with GEM/DOX/MPLA‐L was eventually removed from the study due to weight loss greater than 15% of its starting body weight. However, all remaining mice recovered and did not have significantly different weights from the control group by the end of the study. When tumors were extracted at the end of the study on day 27, GEM/DOX/MPLA‐L showed no tumor mass in six out of eight mice, whereas GEM/DOX‐L led to no detectable tumor mass in only one mouse out of nine. The extracted tumors were weighed, and while both treatment groups had a significantly smaller average mass than the controls, no significant difference could be measured between the treatment groups (Figure 6c). Tumors after extraction are shown in Figure S10, and a direct comparison between the tumor masses of GEM/DOX‐L and GEM/DOX/MPLA‐L is given in Figure S11.

**FIGURE 7:**
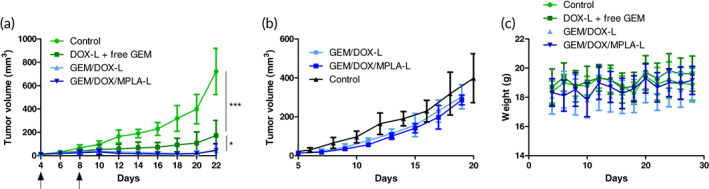
GEM/DOX‐L and GEM/DOX/MPLA‐L were compared in terms of efficacy in a tumor rechallenge study. Two injections of 100 μl of 0.54 mg/ml DOX and 0.28 mg/ml GEM were injected, which translates to 3 mg/kg DOX and 1.55 mg/ml GEM. Mice treated with GEM/DOX/MPLA‐L received 4.3 μg MPLA per injection. (a) Tumor volume was recorded after two injections of DOX‐L and free GEM, GEM/DOX‐L, and GEM/DOX/MPLA‐L with equivalent doses of 3 mg/kg DOX and 1.55 mg/kg GEM. Significance is reported in terms of comparing control group to DOX‐L and free GEM, as well as DOX‐L and free GEM to GEM/DOX‐L. (b) Upon tumor rechallenge of the GEM/DOX‐L and GEM/DOX/MPLA‐L treatment groups, little difference was observed in tumor volume, and the previously measured control group. (c) Mice weight remained consistent throughout the study

To further investigate and evaluate the relevance of MPLA addition into GEM/DOX liposomes, we proceeded with a tumor rechallenge in the opposite mammary fat pad using the 4T1 model in BALB/c mice. As before, treatment occurred when tumors were ~15 mm^3^ in size. However, one notable difference in this study was that two injections of treatment were given to remain consistent with tumor immune profiling conditions. The MPLA content in this experiment was slightly lower at 4.3 μg per injection. GEM/DOX‐L and GEM/DOX/MPLA‐L again both showed very similar tumor volume control (Figure 7a). Upon re‐challenge, tumor growth in both groups was similar (Figure 7b), and there was no weight loss in the MPLA‐treated group due to less aggressive dosing (Figure 7c). The immunogenic cell death of 4T1 cells and enhanced dendritic cell infiltration do not appear to yield long‐term immune memory under the current conditions.

## DISCUSSION

Effective treatment of breast cancer remains a clinical challenge. This is further compounded by the heterogeneity of breast cancer, which can be generalized by the presence of three receptors: estrogen receptor (ER)‐positive, progesterone receptor (PR)‐positive, and human epidermal growth factor receptor 2 (HER2)‐positive. The lack of all three characteristic receptors defines the triple negative subtype of breast cancer, both the most aggressive and immunosuppressive form of breast cancer.^34^ The current standard of care for breast cancer includes aggressive chemotherapy regimens, resection, and radiotherapy. However, it is increasingly shown that the tumor microenvironment immune cell infiltrates play a large role in influencing clinical outcome and patient prognosis.^35^, ^36^ Treatments for breast cancer are rapidly being reconsidered for use with immunotherapy or immunostimulants.

Chemotherapy is traditionally viewed as immunosuppressive, and initially not considered for combination with immunogenic compounds. When dosed at high levels to maximize antitumor cytotoxicity, an unfortunate consequence is the obliteration of immune cell progenitors, leading to severe myelosuppression. However, a large body of literature now documents the immune effects of various classes of chemotherapeutics when not given at the maximum tolerated dosage,^37^, ^38^, ^39^ and several clinical trials have investigated the benefits of adding chemotherapeutics to immunotherapy‐focused regimens.^40^, ^41^, ^42^ Anthracyclines such as DOX induce the characteristic signs of immunogenic cell death by triggering tumor signaling pathways that lead to the upregulation of calreticulin on the cell surface, prompting dendritic cell activation and subsequent antigen presentation.^20^, ^43^ In addition, nanocarriers such as liposomes protect the loaded drug cargo from elimination and prolong circulation half‐life to increase drug bioavailability.^22^ However, DOX liposomes alone were unable to trigger an adaptive immune response in the highly aggressive 4T1 murine breast cancer tumor model.^19^ 4T1 is a form of triple negative breast cancer, which has been shown to have a lower mutational burden than other subtypes of breast cancer. Our approach was to combine MPLA, a potent TLR4 agonist, with a chemotherapeutic combination of DOX and GEM to further amplify the tumor immune response. MPLA has been explored for use in cancer vaccines^44^ but has not been studied extensively in combination with chemotherapy. We used MPLA to enhance the natural immunogenicity of chemotherapeutics in a novel and translatable dual‐loaded liposome with MPLA in the lipid bilayer.

Other studies have confirmed that MPLA can increase the efficacy of DOX liposomes.^13^ However, the separate administration of MPLA microparticles and DOX liposomes inevitably leads to differences in pharmacokinetic profiles, which can reduce the impact of the combination. Additionally, we incorporated a second chemotherapeutic, GEM, as the combination of GEM and DOX has been tested extensively in clinical trials for breast cancer^45^, ^46^ and GEM has been shown to stimulate different anti‐tumor immune responses than DOX.^21^ We pursued a co‐loaded DOX, GEM, and MPLA liposomal formulation to ensure controlled drug ratios and consistent MPLA concentration throughout the circulation time of the formulation. We confirmed the effect of MPLA both in vitro and in vivo, and evaluated the benefits in tumor efficacy that resulted from this combination.

GEM/DOX‐L was shown to increase the ratio of CD80^+^F4/80^+^ (M1) to CD206^+^F4/80^+^ (M2) macrophages. GEM nanoparticles have been documented to skew macrophage polarization toward the M1 phenotype in a melanoma model in C57BL/6 mice^47^ and GEM is acknowledged to deplete myeloid‐derived suppressor cells in breast cancer^48^ and lymphoma models.^49^ However, GEM/DOX‐L did not cause significant activation of dendritic cells, which are essential to mounting an anti‐tumor immune response. GEM/DOX/MPLA‐L treatment did not express significantly higher levels of M1 macrophages than the GEM/DOX‐L‐treated group. The primary confirmed effect of MPLA in GEM/DOX/MPLA‐L was the increase in dendritic cell activation. Dendritic cells are particularly important in mediating the immunogenic cell death process of DOX, as they detect the upregulation of tumor antigens caused by DOX treatment.^20^ In vitro experiments suggest that DOX combined with MPLA provided higher expression of the tumor antigen calreticulin while MPLA stimulated dendritic cell activation to recognize exposed antigens. The in vivo effect of DOX‐initiated immunogenic cell death has been well characterized in the 4T1 tumor model.^43^

GEM/DOX/MPLA‐L was dosed at the same chemotherapeutic drug concentrations as its GEM/DOX‐L counterpart (3 mg/kg DOX, 1.55 mg/kg GEM). However, while animals treated with GEM/DOX‐L displayed no signs of toxicity, GEM/DOX/MPLA‐L appeared to cause more animal weight loss than its GEM/DOX counterpart. Mice injected with GEM/DOX/MPLA‐L received 5.7 μg of MPLA (equating to a 0.3 mg/kg dosage), which falls in the range of MPLA generally used in vaccinations or given intravenously (1–10 μg).^50^ Intravenous administration of MPLA has been given in the range of 0.2–2 mg/kg in C57BL/6 mice.^51^ We hypothesize that the combination with chemotherapy may cause overlapping toxicity profiles, and dosing will need adjustment presumably upon translation to different animal models.

Despite initial immune activation in treated tumors, a tumor rechallenge study with 4T1 cells in the opposite mammary fat pad was not able to produce significant differences in subsequent tumor growth. Based on this, the addition of MPLA was unable to create sustained immune responses. Other treatments involving immunogenic cell death caused by physical cues such as local photodynamic therapy on 4T1 tumors^52^ and local nanopulse stimulation^53^ have shown successful reduction of abscopal tumors. Similarly, after vaccination with irradiated CT26 tumor cells treated with a DOX liposome and microbubble complex, rechallenged tumor growth showed reduced tumor volume compared to vaccination with tumor cells treated with control formulations.^54^ These reported methods demonstrate that treatment‐induced immunogenic cell death can produce long‐lasting immune responses. The lack of long‐term immunity in mice treated with GEM/DOX/MPLA‐L may be related to the dosing regimen. However, more aggressive dosing may lead to toxicity due to the systemic administration of treatment. Future studies should focus on an in‐depth evaluation and optimization of MPLA‐mediated immune activation and its interaction with GEM/DOX‐L. GEM/DOX‐L also proved to be more effective in reducing tumor size than DOX‐L and an equivalent amount of free GEM, which highlights the overall efficacy of the co‐encapsulated GEM/DOX combination.

## CONCLUSION

Unique combinations of chemotherapy and immune‐modulating agents can influence nonimmunogenic tumor environments to create potential targets for immunotherapies. We have shown that the commonly used chemotherapeutic combination of GEM and DOX can influence tumor infiltrating lymphocytes when combined with a potent TLR4 agonist, MPLA, in the aggressive 4T1 tumor model. While tumor volume was comparable, GEM/DOX/MPLA‐L regressed tumors in six out of eight mice at the time of tumor extraction. However, the rechallenge of tumors in both the GEM/DOX‐L and GEM/DOX/MPLA‐L treatment groups were unable to suppress growth of newly implanted tumors, indicating the absence of a long‐lasting immune memory. The heightened immune response during treatment, however, can potentially make GEM/DOX/MPLA‐L an interesting liposomal formulation to pair with immunotherapy in future studies and presents an interesting translational opportunity.

## Supporting information


**Appendix** S1: Supporting Information
