# Supplementary material for: Gemcitabine and doxorubicin in immunostimulatory monophosphoryl lipid A liposomes for treating breast cancer
Source: Bioeng Transl Med. 2020 Sep 16;6(1):e10188. doi: 10.1002/btm2.10188 (PMC7823124; doi:10.1002/btm2.10188)
Supplement: Supplementary file 1 — Appendix S1: Supporting Information [file BTM2-6-e10188-s001.docx]

**Table S1**. Antibodies used in staining cell markers in flow cytometry analysis

| **Antibody** | **Clone** | **Host** | **Fluorophore** | **Supplier** |
| --- | --- | --- | --- | --- |
| CD45 | 30-F11 | Rat | FITC | Thermo Fisher |
| CD11b | M1/70 | Rat | APC |  |
| CD11c | N418 | Armenian hamster | PE |  |
| CD3e | 145-2C11 | Armenian hamster | PerCP-Cy5.5 |  |
| CD4 | GK1.5 | Rat | PE-Cyanine7 |  |
| CD8a | 53-6.7 | Rat | PE |  |
| F4/80 | BM8 | Rat | PerCP-Cyanine5.5 |  |
| CD206 | MMR | Rat | PE-Cyanine7 |  |
| CD80 | B7-1 | Armenian hamster | PE |  |
| Ly-6G | RB6-8C5 | Rat | PE-Cyanine7 |  |
| Calreticulin | EPR3924 | Rabbit | Alexa Fluor 647 | Abcam |
| MHC II | M5/114.15.2 | Rat | PE-Cy7 | Biolegend |
| MHC I | SF1-1.1 | Mouse | BV421 |  |
| CD86 | GL-1 | Rat | APC-Cyanine7 |  |

 **Fig S1. (A)** The mean fluorescence intensity fold increase of calreticulin on 4T1 cells in response to DOX (10 µM) treatment both alone and in combination with blank liposomes and MPLA-L (5 µg) (*n* = 3). Experiment was performed in triplicate wells. Combination with liposomes significantly increased calreticulin exposure. The fold increase of calreticulin is in reference to untreated 4T1 cells. **(B)** Co-culture of 1:1 4T1 and JAWSII cells significantly upregulated co-stimulatory ligand CD40 when treated DOX/MPLA-L (*n* = 3). The fold increase of CD40 is in reference to treatment with an equivalent amount of blank liposomes.


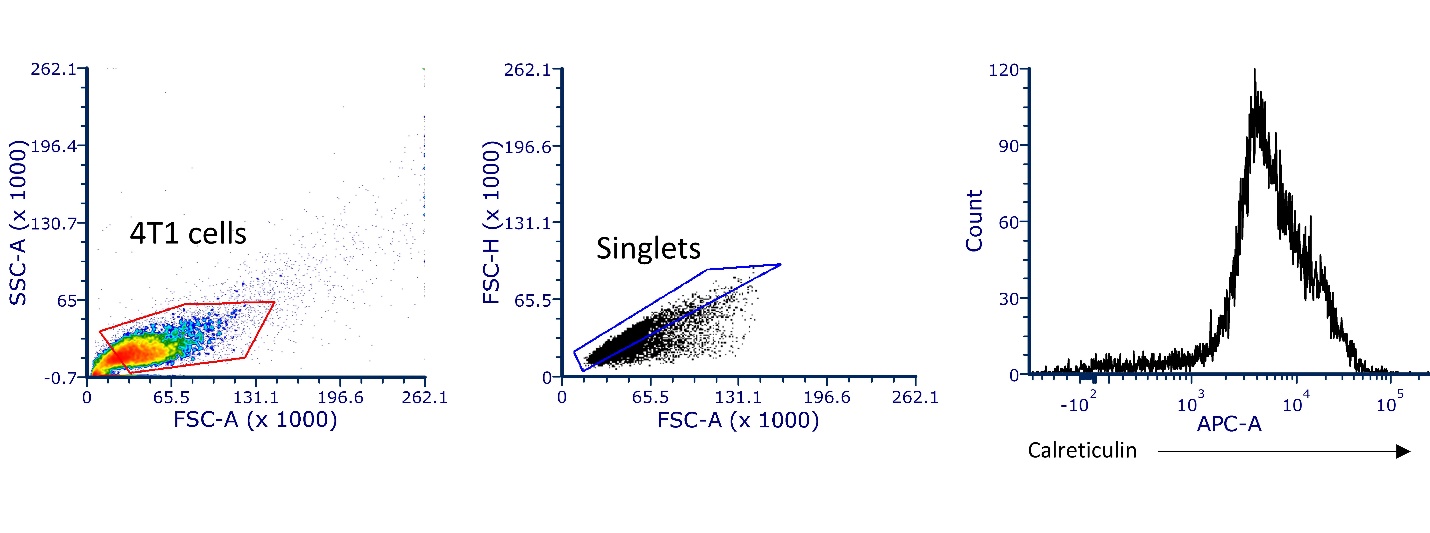
**Fig S2**. Representative flow gating strategy for in vitro experiments involving 4T1 cells.


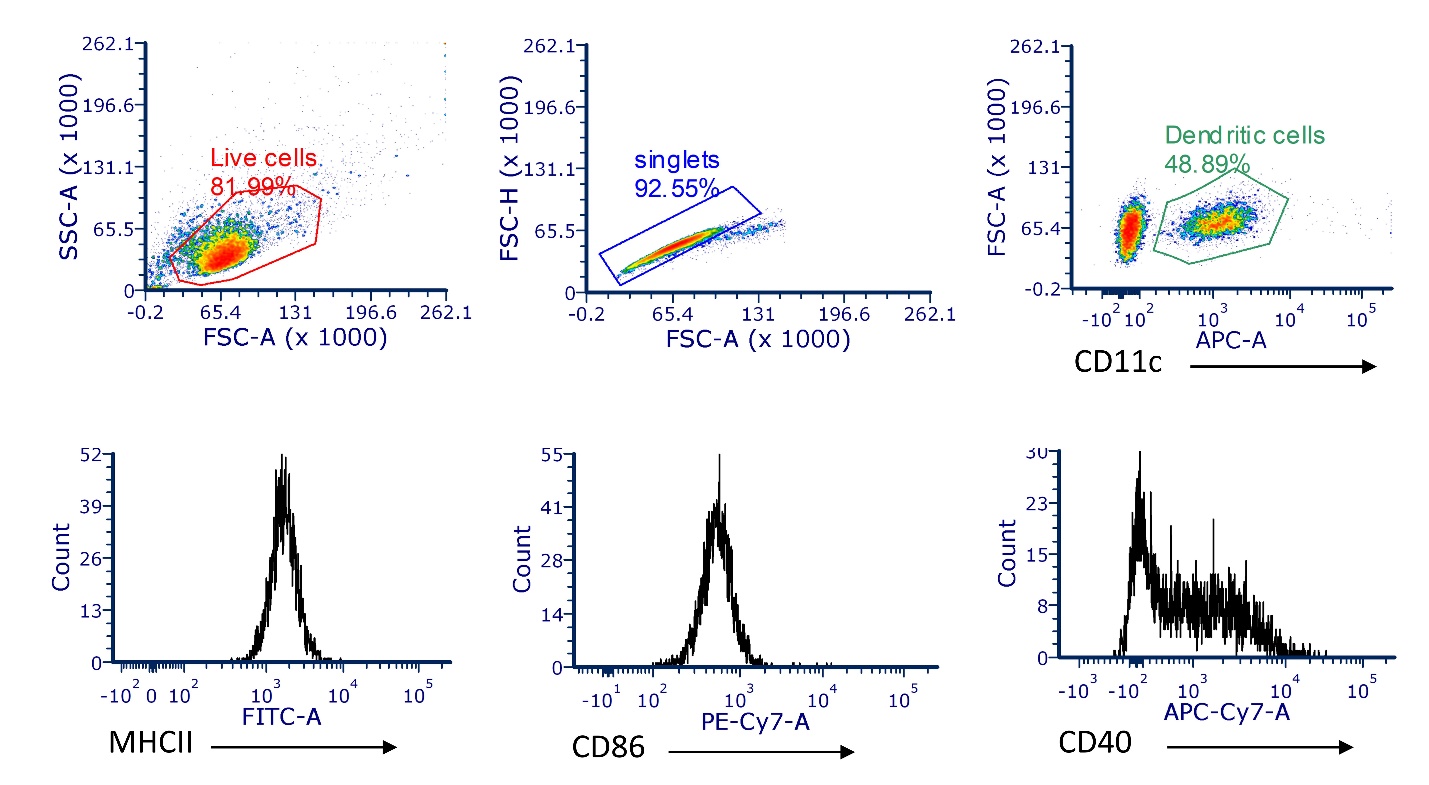


**Fig S3**. Representative gating and analysis of dendritic cells in co-culture with 4T1 cells.

**Fig S4**. Release profile of liposomal formulations over 24 hours at 37°C in PBS. Error bars represent *n* = 5. **(A)** GEM/DOX-L **(B)** GEM/DOX/MPLA-L

**Fig S5**. Immune profiling of 4T1 tumors reveals negligible differences in GEM/DOX-L and GEM/DOX/MPLA-L in regards to **(A)** CD11c^+^CD11b^+^ dendritic cells and **(B)** Ly6G^+^CD11b^+^ MDSCs.


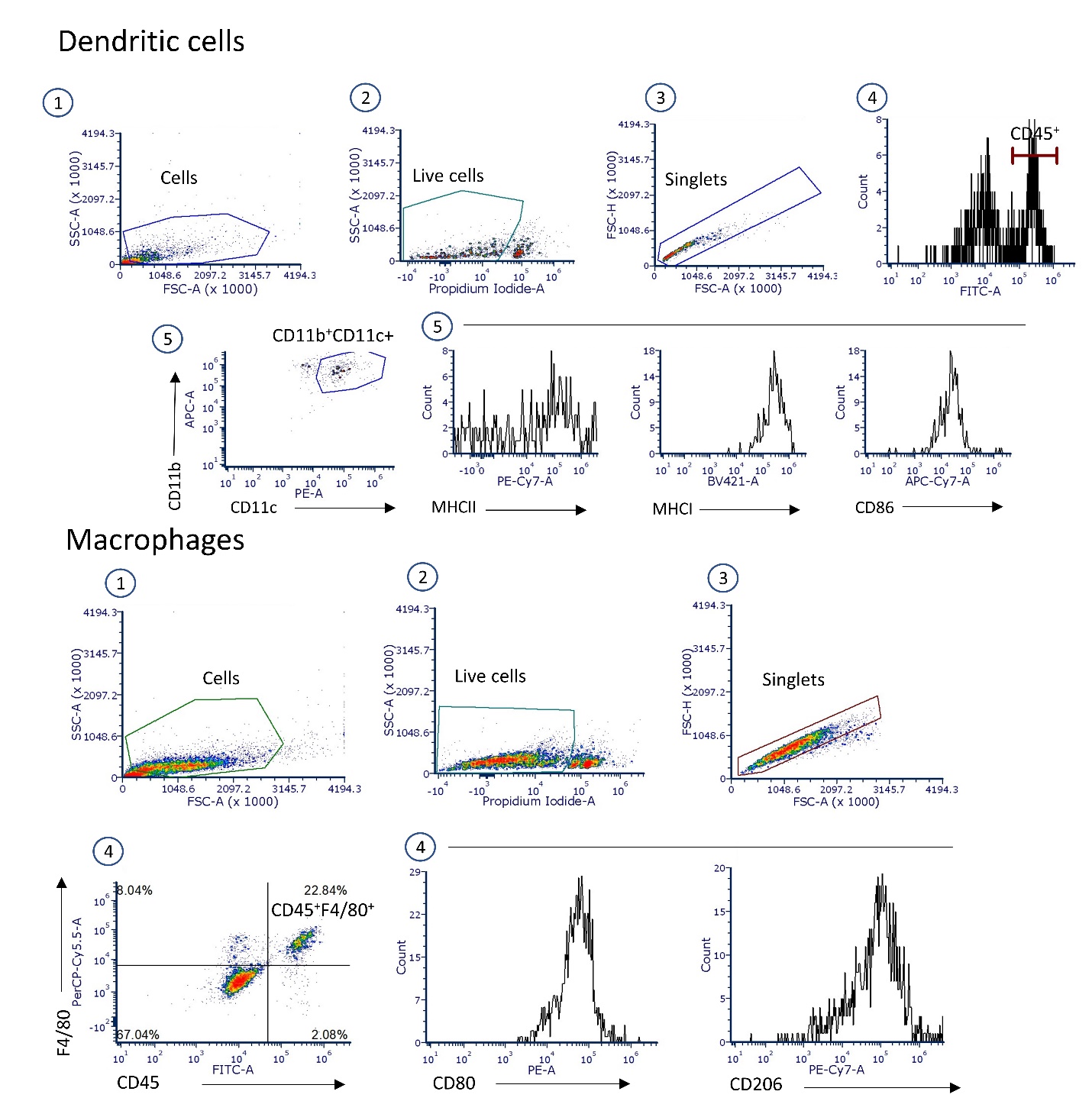


**Fig S6**. Representative gating of dendritic cells and macrophages after tumor extraction and fluorescent antibody staining. Subsequent numbering indicate gates with the previous number as the parent gate.

**Fig S7**. Dendritic cell (Fig 4) and macrophage (Fig 5) population shown as a percentage of total measured cells. **(A)** CD11b^+^CD11c^+^ dendritic cells **(B)** F4/80^+^ macrophages.


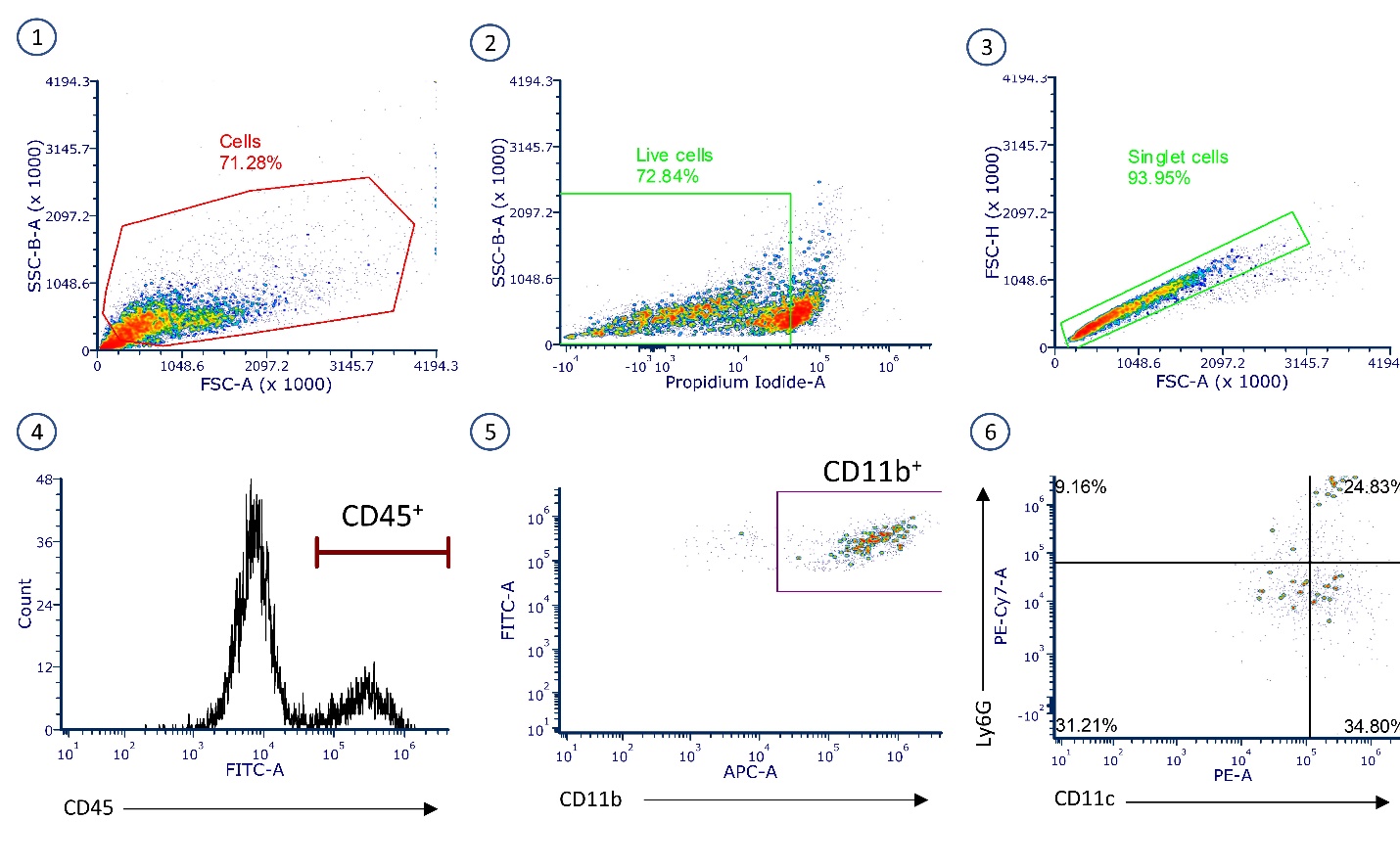


**Fig S8**. Representative gating of dendritic cells and Ly6G^+^ myeloid-derived suppressor cells. Subsequent numbering indicate gates with the previous number as the parent gate.

**Fig S9**. Mass of 4T1 tumors prior to tumor dissociation for immune profiling.


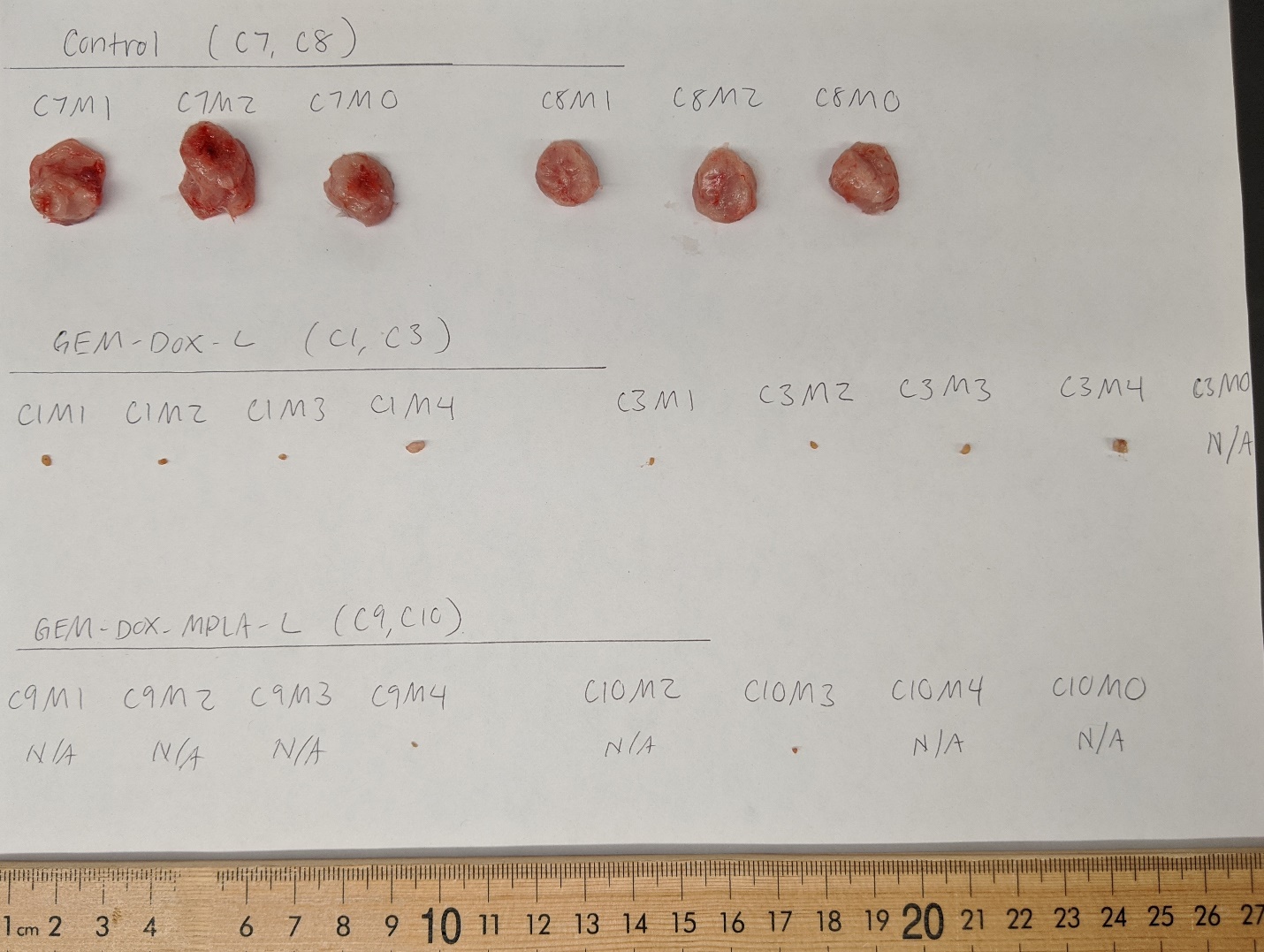


**Fig S10**. Tumors after extraction on day 27 of the efficacy study.

**Fig S11**. Tumor mass comparison between GEM/DOX-L (n = 8) and GEM/DOX/MPLA-L (n = 2) after tumor extraction.
